# The Time-Course of the Last-Presented Benefit in Working Memory: Shifts in the Content of the Focus of Attention

**DOI:** 10.5334/joc.199

**Published:** 2022-01-07

**Authors:** Beatrice Valentini, Kim Uittenhove, Evie Vergauwe

**Affiliations:** 1 University of Geneva, Switzerland; 2 University of Lausanne, Switzerland

**Keywords:** working memory, focus of attention

## Abstract

Working memory is a cognitive system responsible for maintaining information. It is often assumed to contain different states of accessibility of information, which is highest for an item held in the focus of attention. Evidence for this heightened accessibility usually comes from item-recognition tasks, in which a memory list is followed by a probe to be judged as being present in or absent from the list. Probes corresponding to the last-presented list item are usually recognized faster than probes corresponding to any other list item (i.e., the last-presented benefit), an effect that is often explained by the last-presented item being in the focus of attention. The last-presented benefit usually disappears when a long retention interval is inserted between the presentation of the list items and the probe. This raises the question of how long the last-presented item remains in the focus of attention. The present study gradually manipulates the retention interval between the presentation of the list of items and the probe in an item-recognition task in order to pinpoint when the focus of attention switches away from the last-presented list item. The results show that the last-presented benefit decreases over time when the retention interval is gradually extended from 0 ms to 200 ms, 400 ms and 500 ms, and completely disappears as of 750 ms. The cognitive mechanisms that may be involved in the time course of the last-presented benefit are discussed.

## Introduction

Working memory is a limited-capacity cognitive system that is responsible for holding information that is no longer perceptually accessible. Since it underlies a variety of everyday human abilities, such as reasoning and reading comprehension (e.g., Barrouillet & Lecas, 1999; Daneman & Carpenter, 1980; Miyake, Just, & Carpenter, 1994; Kane et al., 2004; Kyllonen & Christal, 1990; Süß et al., 2002), it is important to investigate its limits and the details of its functioning. Several models propose different states in working memory, characterized by different levels of accessibility and different amounts of information that can be maintained (e.g. Cowan, 1988; Cowan, 2011; Nee & Jonides, 2008; Oberauer, 2002; LaRocque et al., 2014; Postle, 2006). Although there is disagreement on the exact number and nature of the different states, many models agree on the existence of a focus of attention (FOA) limited to one item (e.g., McElree, 1998; Nee & Jonides, 2011; Oberauer, 2002). The item in the FOA is assumed to be in a privileged state of heightened accessibility (e.g., Basak & Verhaeghen, 2011; Hitch et al., 2020; Nee & Jonides, 2008; Oberauer, 2002), ready to be the object of the next action or thought. Importantly, this item is assumed to be accessed and processed faster than the items outside of the FOA.

This attribute of heightened accessibility has been demonstrated using the item-recognition task (Sternberg, 1966, 1969), in which participants have to maintain a memory list and decide as quickly as possible whether a subsequent probe belonged to it or not. In this task, the last-presented item of the memory list is often the fastest probe responded to (e.g., Burrows & Okada, 1971; Corbellis, 1967; McElree & Dosher, 1993; Nee & Jonides, 2008), especially when the presentation of the memory list is fast-paced. This last-presented benefit is consistent with the assumed privileged status of the last-presented item due to the item residing in the FOA after list presentation.

However, a recent study showed that a last-presented benefit is not always present. For example, when a retention interval is inserted between the presentation of the list items and the probe, the last-presented benefit is no longer observed (Vergauwe & Langerock, 2017). This finding indicates that the last-presented item is in the FOA right after list presentation (resulting in a last-presented benefit) but that the FOA switches away from the last-presented item at some point (resulting in the disappearance of the last-presented benefit). The current project aims to determine the time-course of the last-presented benefit through the gradual manipulation of the duration of the retention interval between the memory list and the test probe. Understanding the time-course of the last-presented benefit after list presentation will help uncover how long attention lingers on the last-presented item before switching away from the last-presented item, presumably to be used for attention-demanding maintenance processes such as refreshing (Camos et al., 2018) or elaboration (Bartsch, Singmann, & Oberauer, 2018).

## The time-course of the last-presented benefit

Vergauwe and Langerock (2017) presented participants with a list of four to-be-maintained letters followed by a probe letter to be judged as present or absent from the memory list (i.e., the item-recognition task; Sternberg, 1966). Participants had to make their judgment as quickly as possible while minimizing errors. To examine the presence vs. absence of the last-presented benefit, reaction times to probes matching the last-presented item were compared with reaction times to probes matching the other list items. The results showed strong evidence for a last-presented benefit, especially when the memory list was presented at a faster pace (i.e., 1 letter every 350 ms) and immediately followed by a probe such that the time available for post-encoding processes during and after list presentation was severely limited (Vergauwe & Langerock, 2017). This suggests that the last-presented item was still in the FOA immediately after the fast list presentation.

However, when fast list presentation was followed by an empty delay of 1000 ms before the probe, thereby providing some time for post-encoding processes to occur after list presentation, the last-presented benefit disappeared. This indicates that the FOA had switched away from the last-presented letter about 1 second after list presentation, presumably to be used for attention-based maintenance processes. Compatible results can be found in other studies. In fact, the studies with a last-presented benefit often share very short durations of the delay between the memory list and the test probe, i.e., from 0 to 500 ms (e.g., Burrows & Okada, 1971; Corbellis, 1967; McElree & Dosher, 1993; Morin, DeRosa, & Stultz, 1967; Nee & Jonides, 2008), whereas studies with rather long empty delays appear to lead to the disappearance of the last-presented benefit (e.g., Clifton & Birenbaum, 1970; Donkin and Nosofsky, 2012; see ***Table 1***).

**Table 1:** Overview of studies using an item-recognition task, together with the duration of Item presentation and of Delay, and whether or not a last-presented benefit was observed. Studies are listed from the shortest (0 ms) to longest (4800 ms) Delay duration.


AUTHOR	ITEM PRESENTATION	DELAY	LAST-PRESENTED BENEFIT	

Morin, DeRosa, & Stultz, 1967	500 ms	0 ms	Yes	

Monsell, 1978	400 ms	100 ms	Yes	

Vergauwe & Langerock, 2017	250 ms	150 ms	Yes	

McElree & Dosher, 1989	500 ms	300 ms	Yes	

Nee & Jonides, 2008	500 ms	300 ms	Yes	

Corballis, 1967	150 ms	450 ms	Yes	

Burrows & Okada, 1971	500 ms	500 ms	Yes	

Donskin & Nosofsky, 2012	500 ms	600 ms	Yes	

Monsell, 1978	400 ms	600 ms	Yes	

Clifton & Birenbaum, 1970	1500 ms	800 ms	Yes	

Corballis, 1967	300 ms	900 ms	Yes	

Vergauwe & Langerock, 2017	250 ms	1150 ms		No

Donskin & Nosofsky, 2012	1000 ms	2000 ms		No

Burrows & Okada, 1971	1200 ms	2400 ms	Yes	

Clifton & Birenbaum, 1970	1500 ms	2800 ms		No

Clifton & Birenbaum, 1970	1500 ms	4800 ms		No


*Note*: Studies included in the table were item-recognition tasks with sequential item presentation, sub-span memory lists, simple verbal materials, single probes and healthy young adults as participants. Duration delay runs from the offset of the last memory item to the presentation of the probe and thus includes the sum of empty delays, and potential sensory masks or warning images for the test images.

This raises the question of the exact time-course of the last-presented benefit; at what point in time does attention switch away from the last-presented item? Further investigations appear necessary to pinpoint more precisely when the FOA switches away from the last-presented list item, presumably to start post-encoding processes that support working memory maintenance of the list. This paper aims to solve this gap of knowledge.

## The current study

The current paper reports two experiments studying the time-course of the last-presented benefit, with the aim to uncover when the FOA switches away from the representation of the last-presented memory item. Thus, the goal is to pinpoint at what point in time the last-presented benefit disappears, i.e., what delay following stimulus presentation is sufficiently long for the FOA to switch away from the last-presented item. To explore this, we presented a memory list at a fast rate such that the time available for post-encoding processes was severely limited during list presentation, and then carefully manipulated the empty delay that was presented between the last memory item and the presentation of the probe.

The presentation rates used for the to-be-remembered letters have already been shown to bring strong evidence in favour of a last-presented benefit when no delay is provided between the memory list and the test probe (Vergauwe & Langerock, 2017, Experiment 4), consistent with the idea that right after the list is presented very quickly, the last-presented item is still in the FOA. Thus, in Experiment 1, we replicated this condition (i.e., empty delay of 0 ms) as well as three additional time-points (i.e., 500 ms, 1000 ms, and 2000 ms). To anticipate, the findings of Experiment 1 showed that the last-presented benefit disappeared between a delay of 500 and 1000 ms. To pinpoint the exact time point of the attentional shift of interest more precisely, we included some additional time-points in Experiment 2 in approximately that range, such that the probe was presented after an empty delay of 0 ms, 200 ms, 400 ms, 750 ms or 1500 ms in Experiment 2. Note that by providing a spread of delays, including 1500 ms, Experiment 2 can also confirm the generalisability of the findings of Experiment 1 at long delays.

## Methods

### Participants and Design

Two different groups of 36 participants (31 women, mean age = 21.03, SD = 3.3 in Experiment 1, and 28 women, mean age = 20.42, SD =2.0 in Experiment 2) took part in the study. Similar sample sizes were reported in previously published studies using item recognition tasks in our lab (e.g., Uittenhove & Vergauwe, 2019; Vergauwe & Langerock, 2017). All participants were undergraduate students at the University of Geneva and received partial course credit in exchange for their participation., All participants had normal or corrected to-normal vision. Prior to their participation in our experiment, every participant signed an informed consent. Using a repeated-measures design, each participant in Experiment 1 was tested with four different empty delay durations (0, 500, 1000, and 2000 ms) and each participant in Experiment 2 was tested with five different empty delay durations (0, 200, 400, 750, and 1500 ms). The present study was approved by the ethics commission of the Faculty of Psychology and Educational Sciences at the University of Geneva.

### Material and Procedure

**Figure 1:**
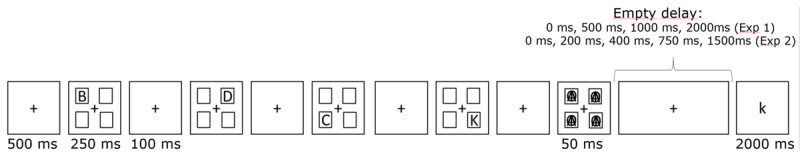
Illustration of the events on a single trial in Experiments 1 and 2.

The task was administered using Tscope5, a C/C++ experiment programming library (Stevens et al, 2006). The program and the materials are all available at *https://osf.io/ngvjk/?view_only=892fa50f9b25460fb2161a1f46c1caa1*). The task required the memorization of a list of four letters, chosen randomly without replacement from a set of 19 consonants (all except W and Y), and the judgement of whether a following probe letter belonged to the list (see ***Figure 1***).

A list of to-be-remembered letters was presented on the screen at a rate of one every 350 ms in Courier New Font, 90 points, upper case. Each letter appeared in the centre of one of four boxes presented on the screen, namely two in the upper part of the screen and two in the lower part. The size of each box was 5.2 cm by 4 cm and each box had a thin, black border line. The boxes were arranged around the centre with a horizontal separation of 1.7 cm, and a vertical separation of 2 cm.

Each presentation trial began with a centrally displayed fixation cross. After 500 ms, the four-boxes pattern appeared and the first to-be-remembered letter was shown in the upper-left box, for 250 ms. Next, the letter and the boxes disappeared and a fixation cross was presented for 100 ms, followed by the four-boxes pattern again and the second to-be-remembered letter which was presented for 250 ms in the upper-right box. This procedure continued until all four letters had been presented. The order of the locations where the to-be-remembered letters were presented were always the same: upper-left, upper-right, lower-left and lower-right, for the memory items 1 to 4, respectively (see ***Figure 1***).

At the end of the presentation phase, each box was filled for 50 ms with a mask created by the superposition of the letters A, O, I, in the same size and font of the memory list items. After a variable empty delay containing only a central fixation cross, the letter probe was displayed in lower case in the centre of the screen until a response was made or 2000 ms had elapsed. The empty delay was manipulated to last 0 ms, 500 ms, 1000 ms or 2000 ms in Experiment 1, and 0 ms, 200 ms, 400 ms, 750 ms or 1500 ms in Experiment 2. It has to be noted that the use of the mask and the use of upper vs. lower case letters aims at the exclusion of any interference from iconic memory as an explanation of the last-presented benefit.

Participants had to respond by pressing the button 1 of the numeric keypad when the probe corresponded to any to-be-remembered letter or the button 2 when the probe was a new letter. The probe corresponded in 1/3 of trials to the last-presented letter (last-presented probe), in 1/3 of trials to any of the presented letters but the last one (not-last-presented probe) and in 1/3 of trials to a random new letter (new probe; i.e., a letter that was not to be remembered on the current trial). This distribution was chosen to optimize the amount of data points per cell.^1^

Participants were instructed to judge the probe as fast as possible without errors. Following their response, the participants were able to start the next trial by pressing the space bar.

In both experiments, trials with the same empty delay duration were presented in separate blocks. In Experiment 1, the order of the blocks was controlled across participants by presenting different block orders for different participant sub-groups. Since the results from Experiment 1 showed no particular differences among these sub-groups, the blocks were presented in a random order for all the participants in Experiment 2, different for each participant (see Supplementary material 1 for details).

Each experiment included 60 trials per duration block, for a total of 240 test trials in Experiment 1 and 300 test trials in Experiment 2. Before the experimental trials, participants received computerized instructions, including a visualization of one trial and six practice trials. The empty delay included in these training trials depended on the order of the blocks for each participant, with the same delay used as that featured in the first block of trials for that participant.

### Performance-based exclusions

The same performance-based exclusions of similar experiments studying the last-presented benefit were applied (Vergauwe et al., 2016, 2018; Vergauwe & Langerock, 2017). Thus, the data of participants whose mean accuracy of their responses to the probes fell below 55% were discarded (1 participant excluded in each experiment). As a result, for both experiments the following analyses include the data of 35 participants.

## Results

**Figure 2:**
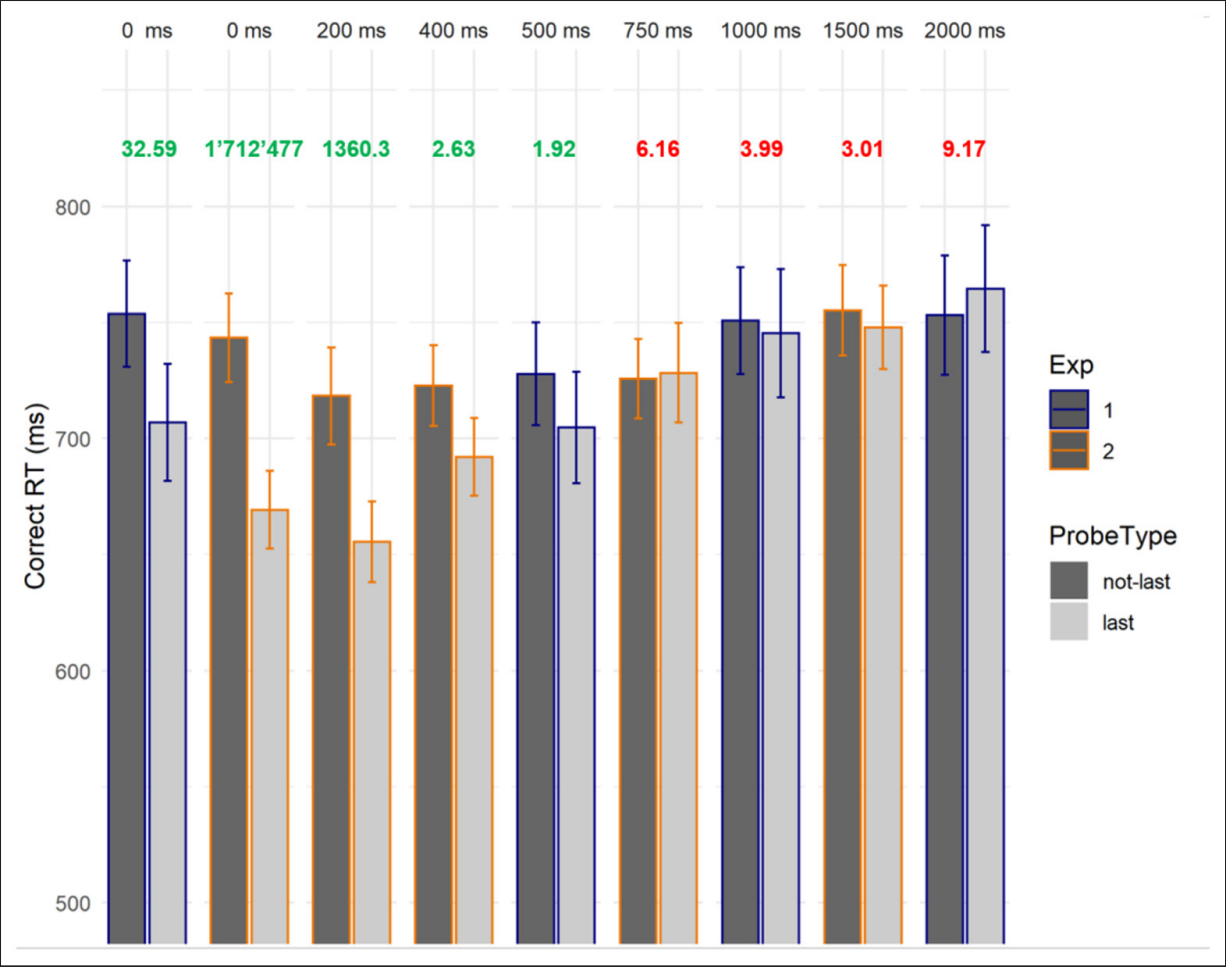
Mean response times in ms for probes matching the last-presented item (“last”) vs. probes matching other list items (“not-last”) in Experiments 1 and 2, together with the evidence in the data (Bayes factors) for (in green) or against (in red) the last-presented benefit in each delay condition. Note that the bars showing data from Experiment 1 have a blue border, while bars showing data from Experiment 2 have an orange border. Error bars represent standard errors of the mean.

Participants had high rates of correct responses to the probes across all delay conditions (89% in Experiment 1 and 90% in Experiment 2; see Supplementary materials 2 and 5 for a detailed breakdown and analysis of accuracy). RT analyses only included correctly responded trials. All analyses were run in R (BayesFactor package), with default settings. For each experiment and for each delay, we assessed the evidence for or against a last-presented benefit (i.e., faster responses for probes matching the last-presented item, compared to other target-presented probes, i.e., not-last-presented probes) with a series of paired, one-sided Bayesian t-tests. In both experiments, there was very strong evidence for a last-presented benefit at the 0 ms delay. Based on Experiment 1, we see that the last-presented benefit disappears between 0 ms and 1000 ms, with anecdotal evidence at 500 ms and substantial evidence against it at 1000 ms. Moreover, Experiment 2 shows substantial evidence against a last-presented benefit from the 750 ms delay (see ***Figure 2***; see Supplementary materials 3 and 4 for a detailed breakdown of RTs and additional analyses).

## Discussion

The current study examined the evidence for or against the last-presented benefit in reaction times to probes presented at different points in time after list presentation. Together, the two experiments show (1) a clear last-presented benefit immediately after fast list-presentation in an item-recognition task, and (2) the rather gradual disappearance of this last-presented benefit when the retention interval after list presentation is gradually extended from 0 to 750 ms. Overall, these results appear to match across-study comparisons showing a last-presented benefit for shorter retention intervals and its absence for retention intervals larger than 1000 ms (see ***Table 1***). Importantly, whereas longer retention intervals were typically associated with slower list presentation, our study held list presentation constant and only manipulated the duration of the following empty delay. It is possible that slower presentation rates may results in a different time-course of the last-presented benefit. Overall, the gradual disappearance of the last-presented benefit suggests that, when giving enough time after list presentation in the current paradigm, the FOA switches away from the last-presented item, thereby removing the accessibility advantage that is present for this memory item at shorter delays.

In this study, we have worked under the assumption that the last-presented benefit reflects the heightened accessibility of the last-presented item residing in the FOA. Working under that assumption, we assumed that the disappearance of the last-presented benefit over time would reflect the FOA switching away from the last-presented item, to start engaging in attention-demanding maintenance processes. There exist several candidates when it comes to the exact nature of these attention-based maintenance processes.

The first possible mechanism is attentional refreshing, i.e. re-activating information by serially cycling attention between items in working memory (e.g., Barrouillet & Camos, 2012; Cowan, 2011; Lisman & Idiart, 1995; Nee & Jonides, 2013; Vergauwe, et al., 2016). When enough free time is given after items presentation, a new refreshing cycle might be initiated by switching the FOA from the last-presented item to the first list item (Barrouillet et al., 2004; Hitch et al., 2020; Vergauwe & Langerock, 2017). Another mechanism could be consolidation, whereby attention strengthens the representation of each to-be-remembered item in order to make them more stable in working memory and thus resistant to forgetting (Arnell, 2006; Jolicoeur & Dell’Acqua, 1998; Ricker & Cowan, 2014; Ricker et al., 2018). This process can explain the reason why a last-presented benefit exists in the first place, i.e., strengthening the last-presented item, and it is consistent with the idea of the completion of consolidation within about 600 ms (e.g., Ricker & Hardman, 2017). Subsequently, attention could be needed to consolidate the rest of the items in a list-wise manner (i.e., list-wide consolidation, see Rhodes & Cowan, 2018). A third option is elaboration, i.e., linking working memory representations into existing semantic networks (e.g., Bartsch, Singmann & Oberauer, 2018; Jonker & Macleod, 2015). This would mean that attention would first encode and process the last-presented letter and then, when additional time is available, it would switch away from the last letter in order to elaborate the other letters. Even if conceptually distinguished from refreshing, elaborative rehearsal might entail refreshing (Bartsch, Singmann & Oberauer, 2018). Finally, a last mechanism that could have been involved is chunking, i.e., gathering different memory items in a single unit (Chen & Cowan, 2005; Portrat et al., 2016; Thalmann, Souza & Oberauer, 2019). According to this account, the last-presented benefit would disappear over time because attention would be used to chunk together the items of the list. Our results cannot distinguish between these different types of attention-based maintenance processes. Furthermore, one cannot exclude the possibility of the FOA switching away from the last-presented item for task-unrelated activities such as mind wandering (e.g., Smallwood & Schooler, 2006).

An alternative view on the last-presented benefit however, is one in terms of higher memory strength or activation of the last-presented item in working memory (e.g., activation-level model in Monsell, 1978, Niklaus et al., 2019), rather than in terms of a qualitatively different state of accessibility of the last-presented item. Under that assumption, it may seem more straightforward to the last-presented item to interpret the disappearance of the last-presented benefit as reflecting the passive decline of the last-item activation. This explanation seems less likely, because if the disappearance of the last presented benefit was passive, we would expect it to occur in all situations and in all ages. Instead, the last-presented benefit seems to not disappear as a function of time in other working memory tasks involving similar periods of time (e.g., the probe span task where probes are presented in between memory items instead of after list presentation; Vergauwe et al., 2016; Vergauwe et al., 2018). Moreover, the last-presented benefit seems not to disappear in school-aged children (Vergauwe et al., 2021), even when much more free time is provided. Accordingly, it seems to be more reasonable to assume that the disappearance of the last-presented benefit in our study reflects something active or strategic.

In conclusion, the present results show a gradual disappearance of the last-presented benefit over time suggesting that, when giving enough time in the current paradigm, the FOA switches away from the last-presented item around 750 ms after list presentation.

## Data Accessibility Statement

The materials, code, and data will be available on OSF (*https://osf.io/ngvjk/?view_only=892fa50f9b25460fb2161a1f46c1caa1*).

## Additional Files

The additional files for this article can be found as follows:

10.5334/joc.199.s1 Supplementary materials 1. Order effects in Experiment 1.

10.5334/joc.199.s2 Supplementary materials 2. Detailed breakdown of accuracy in Experiments 1 and 2.

10.5334/joc.199.s3 Supplementary materials 3. Detailed breakdown of RT in Experiments 1 and 2.

10.5334/joc.199.s4 Supplementary materials 4. ANOVA on RT for the entire dataset.

10.5334/joc.199.s5 Supplementary materials 5. Analysis of accuracy ANOVA for the entire dataset.

## Footnotes

One could argue that this distribution could lead to strategic prioritisation of the last-presented item because it is the most-tested probe (see Atkinson et al., 2018). However, a last-presented benefit has been consistently observed in studies using a uniform distribution across serial positions as well (e.g., Vergauwe et al., 2016, 2018).
