# Supplementary material for: The Time-Course of the Last-Presented Benefit in Working Memory: Shifts in the Content of the Focus of Attention
Source: J Cogn. 2022 Jan 7;5(1):8. doi: 10.5334/joc.199 (PMC8740651; doi:10.5334/joc.199)
Supplement: Supplementary materials 1. — Order effects in Experiment 1. [file joc-5-1-199-s1.pdf]

Supplementary materials 1: Order effects in Experiment 1

In Experiment 1, for one third of participants, the blocks of trials were ordered by increasing delay duration (i.e., Blocks 1 to 4 included 0 ms delay, 500 ms delay, 1000 ms delay, and 2000 ms delay trials, respectively). For another third of participants, blocks were ordered by decreasing delay duration (i.e., Blocks 1 to 4 included 2000 ms delay, 1000 ms delay, 500 ms delay, and 0 ms delay trials, respectively). For the remaining third of participants, the blocks were shown in random order, different for each participant.

In order to test for differences among these three participants sub-groups in Experiment 1, two Bayesian repeated measure ANOVAs were run, one on mean accuracy (i.e., proportion of correct responses) and one on reaction times, both with Order (ascending, descending, orrandom) as between subject variable and ProbeType (last-presented vs. not-last-presented) and Delay (0 ms, 500 ms, 1000 ms, or2000 ms) as within-subject variables. The rationale was to assess the evidence in favour or against the inclusion of a triple interaction between Order, ProbeType and Delay, which could indicate that the effect of Delay on the last-presented benefit differs as a function of the order of the blocks.

The best model for accuracy included Order, ProbeType, and the interaction between Order and ProbeType. Importantly, there was overwhelming evidence against the inclusion of a triple interaction between Order, ProbeType and Delay (BF_{01}= 4412).

For reaction times, the best model included only Delay. There was anecdotal evidence against a main effect of Order (BF_{01}= 2.28), and overwhelming evidence against the inclusion of a triple interaction between Order, ProbeType and Delay (BF_{01} = 50290). Thus, our pattern of interest was not influenced by the order in which the blocks of different delay durations were

presented in Experiment 1. As a result, all participants in Experiment 2 were presented with the blocks in a random way.
