# Supplementary material for: The Time-Course of the Last-Presented Benefit in Working Memory: Shifts in the Content of the Focus of Attention
Source: J Cogn. 2022 Jan 7;5(1):8. doi: 10.5334/joc.199 (PMC8740651; doi:10.5334/joc.199)
Supplement: Supplementary materials 2. — Detailed breakdown of accuracy in Experiments 1 and 2. [file joc-5-1-199-s2.pdf]

Supplementary materials 2: Detailed breakdown of accuracy in Experiments 1 and 2

Table 2: Mean accuracies (percentage correct responses) and standard deviations (in parentheses) for probes matching the last-presented item, probes matching other list items, and new probes, for each delay in Experiments 1 and 2.

	Total accuracy	Last-presented	Not-last-presented	New
0 ms (Exp 1)	88 (6)	92 (8)	84 (11)	88 (10)
0 ms (Exp 2)	91 (6)	95 (6)	89 (10)	88 (8)
200 ms (Exp 2)	91 (5)	96 (5)	92 (12)	86 (8)
400 ms (Exp 2)	90 (6)	94 (7)	91 (10)	86 (8)
500 ms (Exp 1)	89 (8)	93 (9)	83 (14)	90 (9)
750 ms (Exp 2)	92 (5)	95 (4)	95 (9)	87 (6)
1 sec (Exp1)	90 (7)	91 (8)	88 (13)	91 (8)
1.5 sec (Exp 2)	91 (5)	94 (5)	92 (9)	89 (9)
2 sec (Exp 1)	90 (7)	92 (10)	87 (13)	92 (7)

Note: Exp= Experiment
