# Supplementary material for: The Time-Course of the Last-Presented Benefit in Working Memory: Shifts in the Content of the Focus of Attention
Source: J Cogn. 2022 Jan 7;5(1):8. doi: 10.5334/joc.199 (PMC8740651; doi:10.5334/joc.199)
Supplement: Supplementary materials 3. — Detailed breakdown of RT in Experiments 1 and 2. [file joc-5-1-199-s3.pdf]

Supplementary materials 3: Detailed breakdown of RT in Experiments 1 and 2

Table 3: Mean latencies and standard deviations (in parentheses) for probes matching the last-presented item, probes matching other list items, and new probes, for each delay in correct trials only in Experiments 1 and 2.

	Mean latency	Last-presented	Other-presented	New presented
0 ms (Exp 1)	756 ms (135)	707 ms (150)	754 ms (135)	814 ms (141)
0 ms (Exp 2)	739 ms (105)	669 ms (99)	743 ms(113)	812 ms (137)
200 ms (Exp 2)	713 ms (103)	655 ms (103)	718 ms (123)	771 ms (116)
400 ms (Exp 2)	742 ms (90)	692 ms (99)	723 ms (103)	815 ms (113)
500 ms (Exp 1)	748 ms (134)	705 ms (141)	728 ms (131)	814 ms (151)
750 ms (Exp 2)	751 ms (98)	728 ms (127)	726 ms (101)	799 ms (107)
1 sec (Exp 1)	771 ms (143)	745 ms (164)	751 ms (137)	821 ms (157)
1.5 sec (Exp 2)	777 ms (95)	748 ms (106)	755 ms (115)	829 ms (108)
2 sec (Exp 1)	782 ms (148)	765 ms (162)	753 ms (152)	834 ms (166)

Note: Exp= Experiment
