# Supplementary material for: The Time-Course of the Last-Presented Benefit in Working Memory: Shifts in the Content of the Focus of Attention
Source: J Cogn. 2022 Jan 7;5(1):8. doi: 10.5334/joc.199 (PMC8740651; doi:10.5334/joc.199)
Supplement: Supplementary materials 4. — ANOVA on RT for the entire dataset. [file joc-5-1-199-s4.pdf]

Supplementary materials 4: ANOVA on RT for the entire dataset

The following analyses were performed on correct trials only using the exclusion criteria mentioned in the paper.

Experiment 1: A Bayesian repeated measure ANOVA was run on correct reaction times, with ProbeType (last-presented vs. not-last-presented) and Delay (0 ms, 500 ms, 1000 ms, or 2000 ms) as within-subject variables. The best model included only a main effect for Delay. There was anecdotal evidence against the inclusion of the main effect of ProbeType (BF_{01}=1.21) and substantial evidence against the inclusion of both ProbeType and the Delay x ProbeType interaction (BF_{01}=5.22).

Experiment 2:

The same Bayesian repeated measure ANOVA was run on correct reaction times measured in Experiment 2. This time, the best model included the main effects of Delay and ProbeType, as well as the Delay x ProbeType interaction. However, the evidence for including the Delay x ProbeType interaction was weak (BF_{10}=1.95).

Merged experiments:

Finally, a Bayesian repeated measure ANOVA was run on the full dataset. To handle the different number of levels for the factor Delay in Experiments 1 and 2, we created a new Delay condition as follows: a No Delay condition (0 ms conditions from Experiment 1 and Experiment 2), a Short Delay condition (500 ms from Experiment 1, and 400 ms from Experiment 2) and a Long Delay condition (2000 ms from Experiment 1, and 1500 ms from Experiment 2). As such,

the BANOVA had two within-subject variables: ProbeType (last-presented vs. not-last-presented) and Delay (No Delay, Short Delay, or Long Delay) and one between-subject variable: Experiment (Experiment 1 vs. Experiment 2). The best model included the Delay x ProbeType interaction, and the main effects for Delay and ProbeType. Importantly, there was substantial evidence in favour of including the Delay x ProbeType interaction (BF_{10}=6.74).

Thus, overall, it seems that the reaction time data are best explained by an interaction of Delay x ProbeType. This is consistent with the results described in the manuscript.
