# Supplementary material for: The Time-Course of the Last-Presented Benefit in Working Memory: Shifts in the Content of the Focus of Attention
Source: J Cogn. 2022 Jan 7;5(1):8. doi: 10.5334/joc.199 (PMC8740651; doi:10.5334/joc.199)
Supplement: Supplementary materials 5. — Analysis of accuracy ANOVA for the entire dataset. [file joc-5-1-199-s5.pdf]

Supplementary materials 5: Analysis of accuracy ANOVA for the entire dataset

Experiment 1:

We ran a Bayesian repeated measure ANOVA on mean accuracy (i.e., proportion of correct responses), with ProbeType (last-presented vs. not-last-presented) and Delay (0 ms, 500 ms, 1000 ms, or 2000 ms) as within-subject variables. The best model included ProbeType only. There was very strong evidence against the inclusion of Delay (BF_{01}=36.39) and very strong evidence against the inclusion of both Delay and the interaction between Delay and ProbeType (BF_{01}=73.63).

Experiment 2:

The same analysis was performed on accuracy in Experiment 2. This showed that the best model included ProbeType only. There was very strong evidence against the inclusion of Delay (BF_{01}=64.30) and decisive evidence against both Delay and the interaction between Delay and ProbeType (BF_{01}=290.02).

Merged experiments:

Finally, a Bayesian repeated measure ANOVA was run on the full dataset. To handle the different number of levels for the factor Delay in Experiments 1 and 2, we created a new Delay condition as follows: a No Delay condition (0 ms condition from Experiment 1 and Experiment 2), a Short Delay condition (500 ms from Experiment 1, and 400 ms from Experiment 2) and a Long Delay condition (2000 ms from Experiment 1, and 1500 ms from Experiment 2). As such, the BANOVA had two within-subject variables: ProbeType (last-presented vs. not-last-

presented) and Delay (No Delay, Short Delay, or Long Delay) and one between-subject variable: Experiment (Experiment 1 vs. Experiment 2).

The best model included ProbeType only. There was strong evidence against the inclusion of Delay (BF_{01}=14.517) and very strong evidence against including both Delay and the interaction between Delay and ProbeType (BF_{01}=49.21).

Thus, overall, it seems that the accuracy data is best explained by ProbeType alone. This consistent benefit in accuracy for the last-presented item contrasts with the disappearance of the last-presented benefit over time in reaction times. One way to account for this would be to assume that the last-presented benefit on reaction time reflects a mechanism that is distinct from the one reflected in the last-presented benefit on accuracy.
